# Derivation and preliminary validation of an administrative claims-based algorithm for the effectiveness of medications for rheumatoid arthritis

**DOI:** 10.1186/ar3471

**Published:** 2011-09-20

**Authors:** Jeffrey R Curtis, John W Baddley, Shuo Yang, Nivedita Patkar, Lang Chen, Elizabeth Delzell, Ted R Mikuls, Kenneth G Saag, Jasvinder Singh, Monika Safford, Grant W Cannon

**Affiliations:** 1 Department of Medicine, University of Alabama, 510 20th Street South, FOT 805D, Birmingham, AL 35294, USA; 2 Department of Medicine, Birmingham VA Medical Center, 700 19th Street South, Birmingham, AL 35233, USA; 3 Division of Rheumatology, Omaha VA Medical Center, 4101 Woolworth Avenue, Omaha, NE 68105, USA; 4 Division of Rheumatology, University of Nebraska Medical Center, 42nd and Emile, Omaha, NE 68198, USA; 5 George E Wahlen VA Medical Center, 500 Foothill Drive, Salt Lake City, UT 84148, USA; 6 Division of Rheumatology, University of Utah, 30 North 1900 East, SOM4B200, Salt Lake City, UT 84132, USA

**Keywords:** rheumatoid arthritis, comparative effectiveness, administrative claims data, biologic

## Abstract

**Introduction:**

Administrative claims data have not commonly been used to study the clinical effectiveness of medications for rheumatoid arthritis (RA) because of the lack of a validated algorithm for this outcome. We created and tested a claims-based algorithm to serve as a proxy for the clinical effectiveness of RA medications.

**Methods:**

We linked Veterans Health Administration (VHA) medical and pharmacy claims for RA patients participating in the longitudinal Department of Veterans Affairs (VA) RA registry (VARA). Among individuals for whom treatment with a new biologic agent or nonbiologic disease-modifying agent in rheumatic disease (DMARD) was being initiated and with registry follow-up at 1 year, VARA and administrative data were used to create a gold standard for the claims-based effectiveness algorithm. The gold standard outcome was low disease activity (LDA) (Disease Activity Score using 28 joint counts (DAS28) ≤ 3.2) or improvement in DAS28 by > 1.2 units at 12 ± 2 months, with high adherence to therapy. The claims-based effectiveness algorithm incorporated biologic dose escalation or switching, addition of new disease-modifying agents, increase in oral glucocorticoid use and dose as well as parenteral glucocorticoid injections.

**Results:**

Among 1,397 patients, we identified 305 eligible biologic or DMARD treatment episodes in 269 unique individuals. The patients were primarily men (94%) with a mean (± SD) age of 62 ± 10 years. At 1 year, 27% of treatment episodes achieved the effectiveness gold standard. The performance characteristics of the effectiveness algorithm were as follows: positive predictive value, 76% (95% confidence interval (95% CI) = 71% to 81%); negative predictive value, 90% (95% CI = 88% to 92%); sensitivity, 72% (95% CI = 67% to 77%); and specificity, 91% (95% CI = 89% to 93%).

**Conclusions:**

Administrative claims data may be useful in evaluating the effectiveness of medications for RA. Further validation of this effectiveness algorithm will be useful in assessing its generalizability and performance in other populations.

## Introduction

Large administrative claims databases are commonly used to evaluate medication safety [1,2]. These data sources have a number of advantages, including large size, widespread availability, comprehensiveness and high generalizability to the population being studied. These databases typically capture medical diagnoses, procedures, drug utilization, hospitalizations, costs and mortality. The diagnostic and procedural codes are submitted by healthcare providers in the course of clinical care and can be used alone or combined into a more complex algorithm to identify conditions of interest to researchers [3,4]. Algorithms are available to identify a number of safety-related conditions, including hospital-associated infections, myocardial infarction, stroke, gastrointestinal perforation, gastrointestinal bleeding and fractures [5-14]. In validation studies, most of these algorithms have been shown to have high validity compared to a gold standard of medical record review.

Several studies have also confirmed the validity of various coding algorithms to identify arthritis-specific diagnoses and procedures in different medical settings [15-20]. However, the use of administrative data to study the clinical effectiveness of medications for inflammatory arthritis, such as rheumatoid arthritis (RA), has been limited by the lack of a validated algorithm to serve as a proxy for clinical improvement in RA disease activity. Our objective was to derive and test a claims-based algorithm to serve as a proxy for the effectiveness of medications for RA patients.

## Materials and methods

### Eligible patient population

After obtaining Institutional Review Board approval, we used data from a cohort of patients diagnosed with RA by a rheumatologist on the basis of the American College of Rheumatology 1987 criteria [21]. These patients were participants in the longitudinal Department of Veterans Affairs (VA) RA registry (VARA), which has been described elsewhere [22]. All VARA participants provided their written informed consent. VARA contains demographic, clinical and RA-specific information, including the Disease Activity Score using 28 joint counts (DAS28), as assessed by physicians using the DAS28 [23] and the Clinical Disease Activity Index (CDAI) [24], as well as a biorepository with banked DNA, serum and plasma. VARA data have been collected by rheumatologists at 11 VHA facilities throughout the United States since 2003. We linked VARA participants to the Veterans Health Administration's Medical SAS Datasets present in the VHA administrative databases from 2002 to 2010 to obtain medical and pharmacy claims.

Among VARA enrollees, we used claims data to identify eligible individuals in whom a biologic agent had been initiated. Biologics of interest included abatacept, adalimumab, etanercept, infliximab and rituximab. We defined "initiation" as no prior use of that biologic agent during the past 6 months. Eligible participants must have had a baseline VARA visit on the same day or within 1 month of biologic initiation. The date of initiation of the biologic (the index date) defined the start of a 1-year "treatment episode." To confirm that patients were receiving medications through the VA system, eligible individuals must have filled at least one prescription (of any duration) for any oral medication during the 6 to 12 months prior to the index date. Participants must also have had a follow-up VARA visit that occurred at 1 year ± 2 months after the index date. If there was no VARA visit at 1 year, then these treatment episodes were excluded, as there was no clinical gold standard with which to compare the algorithm's performance. VARA data were used only to capture the DAS28, the CDAI and other clinical characteristics measured at the baseline and outcome VARA visits. All other data used for the analysis were abstracted from the administrative claims data.

To test the performance of the effectiveness algorithm and to see whether it was similar for nonbiologic RA treatments, we performed a separate analysis of RA patients enrolled in VARA who were starting leflunomide (LEF), sulfasalazine (SSZ) or hydroxychloroquine (HCQ) and who also had any prior or current use of methotrexate (MTX). New MTX users were not represented in this analysis, because MTX is typically considered an "anchor" drug for RA patients and generally is continued even if the patient's therapeutic response is suboptimal, in contrast to other RA therapies, where the drugs are typically discontinued if they are not effective. Because of similarities in both the descriptive characteristics of the study populations of biologic and nonbiologic disease-modifying anti-rheumatic drug(DMARD) users and the performance characteristics of the effectiveness algorithm between biologic and DMARD treatment episodes, the data are shown throughout for the biologic users as a unique group and for a combined group of new biologic and nonbiologic DMARD users.

### The clinical effectiveness outcome and the effectiveness algorithm

The gold standard for effectiveness was measured at the 1 year VARA visit following the index visit and was defined as DAS28 ≤ 3.2 units (low disease activity (LDA)) or improvement in DAS28 > 1.2 units [25,26]. The gold standard also required that the patient have high adherence to biologic treatment (for example, medication possession ratio for oral or injectable biologic therapy ≥ 80%) (see Table 1 for further details). The purpose of the adherence requirement was to maximize confidence that observed changes in disease activity were more likely attributable to the treatment started on the index date rather than to natural variations in disease activity, switching to a different RA medication after the index date, or other factors.

**Table 1:** Components of the effectiveness algorithm, assessed between the index date and the outcome visit date approximately one year later

Criteria*	Description and implementation
High adherence to index drug (required)	For etanercept, adalimumab and oral medications, must be ≥ 80% adherent to therapy, calculated as a medication possession ratio [38]
	For infliximab, must have received at least the number of infusions expected between the index and outcome visit dates to conform to a schedule of 0, 2, 6 and 14 weeks and every 8 weeks thereafter
	For abatacept, must have received the number of infusions expected between the index and outcome visit dates to conform to a schedule of once-monthly dosing; missing one infusion is permissible
	For rituximab, criterion is not applicable
Biologic switch or add (prohibited)	Between the index and outcome visit dates, patient cannot initiate therapy with a new biologic agent
Addition of a new nonbiologic DMARD (prohibited)	Between the index and outcome visit dates, patient cannot initiate therapy with a new nonbiologic DMARD (methotrexate, sulfasalazine, leflunomide or hydroxychloroquine) that they were not already taking during the 6 months prior to the index date
Increase in biologic dose or frequency (prohibited)	For etanercept and adalimumab, dose escalation of etanercept to 50 mg twice weekly or adalimumab 40 mg once weekly is prohibited
	For infliximab, difference between ending and starting dose, with each dose rounded up to the nearest 100 mg cannot be ≥ 100 mg. The number of infusions must be within 120% of the number expected assuming a 0-, 2-, or 6-week load and an 8-week infusion schedule
	For abatacept, difference between ending and starting dose cannot be ≥ 100 mg
	For rituximab, criterion is not applicable
More than one glucocorticoid joint injection (prohibited)	Cannot receive glucocorticoid injections† on more than one unique calendar day between the index date + 90 days and the outcome visit date, inclusive
Increase in dose of oral glucocorticoid (prohibited)	For patients who received no prescriptions for oral glucocorticoids during the 6 months prior to the index date, cannot have received more than 30 days of oral glucocorticoids between the index date + 90 days and the outcome visit date, inclusive
	For patients who received prescriptions for oral glucocorticoids in the 6 months prior to the index date, the cumulative glucocorticoid dose in the 6 months prior to the outcome visit date must be similar (that is, within 120%) to the cumulative dose in the 6 months prior to the index visit date

DMARD: disease-modifying agent in rheumatic disease. †Glucocorticoid injection CPT codes: 20600, 20605, 20610. *All criteria must be satisfied to have met the effectiveness algorithm.

The claims-based effectiveness algorithm described in Table 1 incorporates factors (selected *a priori *based upon content knowledge) that were expected to be associated with suboptimal clinical response and would be available within typical administrative claims data sources without laboratory results. The components of the effectiveness algorithm included increase in biologic dose compared to the starting dose; switch to a different biologic; addition of a new nonbiologic DMARD, including MTX, SSZ, LEF and HCQ; initiation of chronic glucocorticoids (for those with no oral glucocorticoid prescriptions during the 6 months prior to the index date); increase in glucocorticoid dose during months 6 to 12 (for those who received any oral glucocorticoid prescriptions in the 6 months prior to the index date); and more than one parenteral or intraarticular injection on unique days after the patient had been receiving the new treatment for more than 3 months. Each of these factors was included in the algorithm as a series of dichotomous conditions that were either satisfied or not. Patients must have satisfied all conditions to have met the effectiveness rule.

### Statistical analysis and additional sensitivity analyses

We calculated the performance characteristics, including positive predictive value (PPV), negative predictive value (NPV), sensitivity (Se) and specificity (Sp), to compare the effectiveness algorithm to the effectiveness gold standard, and we used the binomial distribution to calculate 95% confidence intervals. Because patients were allowed to contribute multiple treatment episodes, we performed an additional analysis where all patients were permitted to contribute only one treatment episode each. This approach was felt to be more conservative than alternate strategies, such as using generalized estimating equations that account for the within-person variance by widening the confidence intervals of the PPV, NPV, Se and Sp, but leave the point estimates unchanged.

For all treatment episodes where there was discordance between the administrative data-based effectiveness rule and the gold standard for clinical effectiveness, we abstracted additional data from the medical records using a structured case report form developed to descriptively inform the reason for discordance.

Although not explicitly part of the effectiveness rule, we also identified comorbidities (posttraumatic stress disorder, low-back pain, fibromyalgia, hepatitis C and depression) that were hypothesized to be associated with worse patient global scores independently of RA disease activity. As part of a sensitivity analysis, we restricted the cohort to patients without any of these ICD-9 codes. As part of two additional sensitivity analyses, we dropped the requirement that patients have a baseline VARA visit. This allowed for inclusion of a modest number of additional VARA treatment episodes where only an outcome VARA visit (but not a baseline VARA visit) was available. In these sensitivity analyses, clinical effectiveness was defined by low disease activity as (1) DAS28 ≤ 3.2 with high adherence or (2) CDAI < 11 with high adherence. All analyses were performed using SAS 9.2 software (SAS Institute, Cary, NC, USA).

## Results

The characteristics of the VARA participants were measured at the start of each treatment episode. Because the characteristics of VARA patients at the start of nonbiologic DMARD treatment episodes were similar to those of the biologic treatment episodes, these data were pooled and are shown in Table 2 as biologic treatment episodes (left column) and a combined group of biologic or nonbiologic DMARD treatment episodes (right column). As shown, and consistent with expectations for this RA population of US veterans [27], 94% were male, the majority were Caucasian and there was a high prevalence of current or past smoking. The most commonly initiated biologic was adalimumab (38%). For all eligible biologic treatment episodes (*n *= 197), patients had high starting disease activity as evidenced by a mean DAS28 of 5.0, a mean tender joint count of 9.6 and a mean swollen joint count of 7.9. After combining the biologic treatment episodes with the DMARD treatment episodes (*n *= 305 total), the descriptive characteristics of the eligible cohort remained similar (right column in Table 2).

**Table 2:** Baseline characteristics of VARA participants at the start of each biologic treatment episode

Characteristics	Biologics only (*N *= 197)	Biologics or DMARDs* (*N *= 305)
Patient demographics		
Age, years	60.9 ± 10.3	62.3 ± 10.4
Males	185 (94%)	287 (94%)
Race/ethnicity		
Caucasian, non-Hispanic	159 (81%)	248 (81%)
Non-Caucasian, Hispanic	7 (4%)	8 (3%)
Black, non-Hispanic	27 (14%)	45 (15%)
American Indian or Pacific Islander	4 (2%)	4 (1%)
RA drug initiated		
Abatacept	9 (5%)	9 (3%)
Adalimumab	74 (38%)	74 (24%)
Etanercept	60 (31%)	60 (20%)
Infliximab	34 (17%)	34 (11%)
Rituximab	20 (10%)	20 (7%)
Hydroxychloroquine	n/a	63 (21%)
Leflunomide	n/a	20 (7%)
Sulfasalazine	n/a	25 (8%)
RA-related characteristics		
DAS28	5.0 ± 1.5	4.9 ± 1.6
CDAI (0-76)	30.2 ± 16.3	27.5 ± 15.2
Physician global (0 to 100)	51.0 ± 22.1	50.3 ± 22.6
Patient global (0 to 100)	57.4 ± 25.2	54.8 ± 24.2
Tender joint count (0 to 28)	9.6 ± 8.6	8.5 ± 7.9
Swollen joint count (0 to 28)	7.9 ± 7.2	7.8 ± 6.6
MDHAQ (0 to 3)	1.2 ± 0.6	1.2 ± 0.6
ESR, mm/hour	27.9 ± 23.3	29.9 ± 24.6
CRP, mg/dL	1.9 ± 2.4	2.1 ± 2.5

Data are *n *(%) or means ± SD. DMARD: disease-modifying agent; RA: rheumatoid arthritis; CDAI: Clinical Disease Activity Index; CRP: C-reactive protein; DAS28: Disease Activity Score in 28 joints; MDHAQ: Multi-Dimensional Health Assessment Questionnaire; ESR: sedimentation rate; n/a: not applicable; SD: standard deviation. *Includes hydroxychloroquine, leflunomide and sulfasalazine.

The primary results of the study are shown in Tables 3 and 4. Among patients treated with biologics (Table 3), a total of 28% of treatment episodes were deemed effective based upon the patients' remaining on therapy and achieving either low disease activity (mean DAS28 ≤ 3.2) and/or a 1.2 unit improvement in DAS28. The PPV and NPV of the administrative data-based effectiveness algorithm were 75% and 90%, respectively. The sensitivity of the effectiveness algorithm was 75%, and its specificity was 90%. If patients were restricted to contributing only one treatment episode (*n *= 161 unique patients), the PPV was 76% and the NPV was 91%.

**Table 3:** Comparison of effectiveness algorithm versus effectiveness gold standard for biologic users

	Effectiveness gold standard*				
Met effectiveness algorithm**	Yes	No	Total	PPV (95% CI)	NPV (95% CI)
Yes	42	14	56 (28%)	75% (62 to 86)	
No	14	127	141 (72%)		90% (84 to 94)
Total	56 (28%)	141 (72%)	197 (100%)		
	Se 75% (95% CI = 62 to 86)	Sp 90% (95% CI = 84 to 94)			

CI: confidence interval; NPV: negative predictive value; PPV: positive predictive value; Se: sensitivity; Sp: specificity. *DAS28 ≤ 3.2 or DAS28 improvement by > 1.2 units and high adherence (for example, ≥ 80%) to the biologic started on the index date. **The components of the effectiveness algorithm are shown in Table 1.

**Table 4:** Comparison of effectiveness algorithm versus effectiveness gold standard for biologic and nonbiologic disease-modifying agent in rheumatic disease** treatments

	Effectiveness gold standard***				
Met effectiveness algorithm*	Yes	No	Total	PPV (95% CI)	NPV (95% CI)
Yes	60	19	79 (26%)	76% (71 to 81)	
No	23	203	226 (74%)		90% (88 to 92)
Total	83 (27%)	222 (73%)	305 (100%)		
	Se = 72% (95% CI = 67 to 77)	Sp = 91% (95% CI = 89 to 93)			

CI: confidence Interval; DMARD: disease-modifying; NPV: negative predictive value; PPV: positive predictive value; Se: sensitivity; Sp: specificity. *The components of the effectiveness algorithm are given in Table 1. **DMARDs include hydroxychloroquine, leflunomide and sulfasalazine. ***Defined as DAS28 ≤ 3.2 or DAS28 improvement by > 1.2 units and high adherence (for example, ≥ 80%) to the biologic and/or DMARD started on the index date.

Among the biologic users in Table 3, the most common reasons why patients failed to meet the effectiveness algorithm criteria were suboptimal adherence, discontinuation and/or switching to a different biologic agent (*n *= 118, 60%); glucocorticoid dose increase (*n *= 30, 15%); addition of new nonbiologic DMARDs (*n *= 23, 12%); biologic agent dose increase (*n *= 15, 8%); glucocorticoid initiation (*n *= 10, 6%); and more than one joint injection (*n *= 11, 6%). The results of the sensitivity analysis that excluded biologic treatment episodes for patients with any of the several comorbidities of interest (33%, *n *= 131 treatment episodes remaining) yielded a slightly higher PPV (81%) and a similar NPV (89%) compared to the main analysis.

The performance characteristics of the combined cohort that included both biologic and nonbiologic treatment episodes are shown in Table 4 and were generally quite similar to the PPV and NPV shown for the biologic treatment episodes in Table 3. Further details obtained from medical record review were available for the patients in the off-diagonal (discordant) cells given in Table 4 and are shown in Table 5. For the 19 treatment episodes where the effectiveness algorithm criteria were satisfied but the gold standard criteria were not, the most common reasons found were either that an inadequate clinical response was recognized but medication changes were precluded because of new or worsened comorbidities, or the physician and/or the patient was satisfied with the level of disease activity, even though the patient did not meet the DAS28 criteria for low disease activity or improvement. For the 23 treatment episodes in which the effectiveness algorithm criteria were not satisfied but the gold standard criteria were, the most common reasons were an increase in the dose of oral glucocorticoids and the addition of new nonbiologic DMARDs.

**Table 5:** Reasons for discordance between the effectiveness algorithm and the effectiveness gold standard

Reasons for discordance	Satisfied effectiveness algorithm, did not meet effectiveness gold standard (false-positives) (*n *= 19)	Did not satisfy effectiveness algorithm, met effectiveness gold standard (false-negatives) (*n *= 23)
Presumed reasons for not meeting gold standard, obtained from medical record review		
Biologic change deferred in light of concerns for new/worsened comorbidity	10	-
Clinically stable or improved and patient/physician satisfied, but DAS and DAS change did not meet gold standard effectiveness criteria	4	-
Physician recognized inadequate response, but chose to retreat with rituximab only after 1 year	2	
Receiving some medications (for example, glucocorticoids) outside of the VHA system	1	-
Biologic change deferred in light of surgery or procedure	1	-
Physician recommended biologic change or dose change, but patient declined	1	-
Noncompliance with nonbiologic RA medications	1	-
Components of the effectiveness algorithm that were not met despite having met the effectiveness gold standard		
Glucocorticoid dose increase or initiation	-	15
Added new DMARD(s)	-	6
Increase in biologic dose and/or frequency	-	2

VHA: Veterans Health Administration; RA: rheumatoid arthritis; DMARD: disease-modifying. Data shown are the number of treatment episodes in the off-diagonal cells given in Table 4. Column totals may sum to > 100% because there may be multiple reasons why patients did not meet the effectiveness gold standard or the effectiveness algorithm. -, criterion is not applicable.

The extent of bias resulting from misclassification of our algorithm is described in Table 6. After varying a hypothetical response rate as measured by the algorithm from 30% and 60%, the amount of bias compared to the true response rate ranged from 1% to 21%.

**Table 6:** Extent of bias associated with misclassification* of the effectiveness algorithm according to observed response rate

Observed response rate	True response rate**	Bias (observed-true)/true (%)
30%	30%	< 1%
40%	36%	10%
50%	43%	16%
60%	49%	21%

*From Table 4, where the positive predictive value (PPV) was 76% and the negative predictive value (NPV) was 90%. **Computed as True rate = Observed rate × (PPV + NPV-1)-NPV + 1 [39]. Numbers are rounded to the nearest whole integer but the actual values were used to calculate the bias.

The results of the second sensitivity analysis that had no baseline VARA visit (and thus could not include change in disease activity as part of the effectiveness gold standard) but included all patients, regardless of comorbidities, are shown in Additional file 1. Many more treatment episodes were available (*n *= 380 for biologic treatment episodes and *n *= 699 for biologic or DMARD treatment episodes). Approximately 20% of patients achieved the effectiveness gold standard, which in this analysis was low disease activity (DAS28 ≤ 3.2). The NPV of the effectiveness algorithm was high (92%), but the PPV was substantially lower (49%). After substituting CDAI < 11 for DAS28 ≤ 3.2 as the gold standard for clinical effectiveness in the third sensitivity analysis, the results were nearly identical (data not shown).

## Discussion

We developed a novel, administrative data-based clinical effectiveness algorithm for use in future studies as a proxy for the clinical effectiveness of RA medications. In this preliminary assessment of its performance, we showed that it has acceptable sensitivity, specificity, PPV and NPV. Our sensitivity, specificity, PPV and NPV that were in the 75% to 90% range reflect good, although not perfect, performance of our effectiveness algorithm applied to administrative claims data. By way of comparison, the corresponding performance characteristics of administrative data for a number of rheumatology conditions, including diagnoses for RA, spondyloarthropathies, systemic lupus erythematosus, fibromyalgia, osteoarthritis, joint injection and joint replacement procedures [15-20] were similar and ranged from approximately 80% to 95%. Besides a new or worsened comorbidity, the most common reason why patients met the effectiveness algorithm criteria but failed to meet the gold standard criteria was that the physician and patient were satisfied with the level of disease activity, despite not having achieved low disease activity or an improvement in the DAS28 by ≥ 1.2 units. In this circumstance, providers may feel that the patient is getting at least some benefit from the drug and that the clinical response is adequate to continue its use. It is also possible that quantitative disease activity measures such as the DAS28 may not adequately capture underlying RA disease activity for some patients (for example, those with concomitant fibromyalgia). Moreover, patients may fear that their condition will worsen after switching to a new therapy or may have trepidation regarding new side effects [28], and therefore they may be reluctant to change medications. Further studies are needed to validate the effectiveness algorithm in other data sets and RA patient populations. However, these results are encouraging and suggest that administrative data can be used to estimate medication effectiveness for RA patients.

As our gold standard for medication effectiveness, we selected low disease activity (DAS28 ≤ 3.2) or improvement in DAS28 by > 1.2 units. It might be argued that these criteria are not stringent enough, although they are broadly consistent with (albeit not identical to) the European League Against Rheumatoid Arthritis (EULAR) responder definition [26]. Consistent with our focus on the DAS28, results from a preference analysis found that RA disease activity score (also measured using the DAS28) was the most important factor in rheumatologists' decisions to escalate care [29]. The results from the Consortium of Rheumatology Researchers of North America (CORRONA) registry showed that low disease activity or a DAS28 improvement > 1.2 units was sufficient for the majority of patients to continue treatment with biologic therapy [30]. As part of a sensitivity analysis, we modified our gold standard to require patients to achieve only LDA (DAS28 ≤ 3.2) and did not include patients who achieved only some improvement (change in DAS28 ≥ 1.2) in the absence of LDA. This lowered the PPV, indicating that many patients had clinical improvement but did not achieve LDA. Many of these patients were continued on therapy, suggesting that both the patients and physicians were in many cases satisfied enough with the response. We also note that the DAS28 response rate (approximately 30%) (Table 3) observed for our clinical effectiveness gold standard was relatively low. However, given the comorbidity profile and other characteristics of the RA patients enrolled in VARA [31], response rates are typically lower than those reported in clinical trials of more selectively included RA patients with fewer comorbidities [32].

Another component of our gold standard is that we required that patients have high (that is, ≥ 80%) adherence to their medication regimen. We recognize that any threshold for adherence is arbitrary. Requiring ≥ 80% compliance is conventional and has been used when studying other conditions, such as osteoporosis and cardiovascular disease [33-36]. The main purpose of the adherence requirement was to focus on medication effectiveness. Medications that the patient does not continue, whether for reasons of inefficacy, safety, tolerability or something else, are not effective. Adherence has been required in other observational analyses of comparative effectiveness in RA [37]. Also, we wanted to maximize confidence in the patient's disease activity's being attributable to the RA treatment started on the index date rather than on a medication that was later substituted because the previous medication begun on the index date had failed. Finally, the requirement of continued adherence to the RA therapy is consistent with clinical trial methodology in which patients who do not adhere to the study protocol, including continuing to take the medication, are generally excluded from the trial. These patients' outcomes are often imputed as nonresponse, which is the same classification to which they were assigned in our effectiveness algorithm.

Although many of the elements of our effectiveness algorithm are intuitive, a few deserve special mention. The requirement that patients not initiate or escalate the dose of oral glucocorticoids assumes that the dominant prescribing indication for glucocorticoids is RA. For patients who may have another indication for glucocorticoids (for example, chronic obstructive pulmonary disease, which is very common in VHA patients), this criterion may not perform optimally. As described in Table 5, this issue was the most common reason why patients failed the effectiveness algorithm. Our algorithm might be expected to perform better in other RA populations that have been shown to have a lower prevalence of comorbidities for which systemic glucocorticoids are used [31]. We also limited the number of intraarticular injections allowable to no more than 1 unique day on which the patient received such injections. VA physicians are not directly compensated for these injections and other procedures and therefore are likely to underreport them. For this reason, our effectiveness algorithm may perform better when there is a financial incentive to code these procedures more accurately. We also found certain comorbidities (for example, fibromyalgia and depression) were common, and we hypothesized that they might be associated with high patient global scores even if the patient's RA is under good control. This is not a unique feature of the VARA cohort or our study, but is potentially problematic for the measurement of patient-reported outcomes in all RA studies that include patients with these conditions. Restricting the population to individuals without these comorbidities improved the PPV of our effectiveness algorithm by 6%, but limits our study's generalizability as it excluded one-third of our data.

The strengths of our study include evaluation of a large number of patients participating in a RA registry at 11 VA medical centers. All patients had rheumatologist-confirmed RA and well-characterized measures of RA disease activity. The novel linkage between the registry and the national VHA administrative data made developing and testing of our effectiveness algorithm possible. Additionally, there are strong financial incentives for RA patients to fill their biologic medications within the VHA system, and it is likely that most if not all RA medications were captured in the VHA administrative data. Despite these strengths, we acknowledge the potentially limited generalizability of patterns of care in the VHA system, and the possible dissimilarity in the RA patients who receive treatment in that system, compared to other RA populations. However, sensitivity andspecificity, unlike PPV and NPV, should be less dependent on the prevalence in the population, and more reflective of the test itself, thereby decreasing the impact of any unique features of the VA population. Moreover, we might expect that the PPV and NPV of the algorithm might perform better in other RA cohorts, given the higher prevalence of comorbidities in this VARA population compared to other RA cohorts [31]. We also acknowledge that while the effectiveness algorithm, which was based upon factors selected from content knowledge, appeared to perform well and have good face validity in VARA, further validation in more recently recruited VARA participants who were not included in our sample, and in different RA cohorts where there is a link to administrative data, is needed to confirm our algorithm's robustness. We also recognize that using more empirical approaches to let the data guide optimization of the algorithm would be desirable, but substantially more data would be required for this approach and for validation. Finally, as an additional opportunity to extend the algorithm in the future, we note that our effectiveness outcome was measured at 1 year, and assessing effectiveness at other time points (for example, at 6 and 24 months) is important. Although we expect similar performance of the algorithm at these different time points, this hypothesis remains to be confirmed.

## Conclusions

In conclusion, the results of this work provide a preliminary mechanism with which to evaluate the effectiveness of RA medications on the basis of administrative claims and pharmacy data. While clinical disease activity measures remain the gold standard for assessing effectiveness in RA, the many large administrative data sources in the United States and internationally are an as yet untapped resource that might be used to assess effectiveness in large real-world populations of RA patients.

## Abbreviations

CDAI: Clinical Disease Activity Index; CORRONA: Consortium of Rheumatology Researchers of North America; DAS28: Disease Activity Score using 28 joint counts; DMARD: disease-modifying anti-rheumatic drug; DSS: Decision Support System; EULAR: European League Against rheumatism; GEE: generalized estimating equation; LDA: low disease activity; MPR: medication possession ratio; NPV: negative predictive value; PPV: positive predictive value; RA: rheumatoid arthritis; Se: sensitivity; Sp: specificity; VARA: Veterans Affairs Rheumatoid Arthritis; VHA: Veterans Health Administration.

## Competing interests

JRC has performed research and consulting for Roche, Genentech, UCB, Abbott, Amgen, CORRONA, Centocor and Bristol-Myers Squibb. All other coauthors have nothing to disclose.

## Authors' contributions

All authors made substantial contributions to the study's conception and design and to the analysis and interpretation of the data. TRM and GWC handled the acquisition of data. All authors contributed to the manuscript revision process and addressed important intellectual content. All authors read and approved the final manuscript for publication.

## Supplementary Material

Additional file 1 **Sensitivity analysis comparing the effectiveness algorithm to an alternate definition of the effectiveness gold standard**. Table S1 Sensitivity analysis comparing the effectiveness algorithm to an alternate definition of the effectiveness gold standard for biologic users. Table S2 Sensitivity analysis comparing the effectiveness algorithm to an alternate definition of the effectiveness gold standard for biologic and nonbiologic disease-modifying agent in rheumatic disease treatments. Click here for file
