# Supplementary material for: Derivation and preliminary validation of an administrative claims-based algorithm for the effectiveness of medications for rheumatoid arthritis
Source: Arthritis Res Ther. 2011 Sep 20;13(5):R155. doi: 10.1186/ar3471 (PMC3308085; doi:10.1186/ar3471)
Supplement: Additional file 1 — Sensitivity analysis comparing the effectiveness algorithm to an alternate definition of the effectiveness gold standard. Table S1 Sensitivity analysis comparing the effectiveness algorithm to an alternate definition of the effectiveness gold standard for biologic users. Table S2 Sensitivity analysis comparing the effectiveness algorithm to an alternate definition of the effectiveness gold standard for biologic and nonbiologic disease-modifying agent in rheumatic disease treatments. [file ar3471-S1.DOCX]

**Appendix Table B: Sensitivity Analysis Comparing the Effectiveness Algorithm* to a Different Definition of the Effectiveness Gold Standard** for Biologic Users**

|  | **Met Effectiveness Gold Standard** (DAS28 <= 3.2)** | | | |  |
| --- | --- | --- | --- | --- | --- |
| **Met Effectiveness Rule** |  | **Yes** | **No** | **Total** |  |
|  | **Yes** | 61 | 52 | 113 (30%) | **PPV = 54%,  95% CI 45, 63%** |
|  | **No** | 18 | 249 | 267 (70%) | **NPV = 93%,**  **95% CI 90, 96%** |
|  | **Total** | 79 (21%) | 301 (79%) | 380 (100%) |  |
|  |  | **Sn = 77% 95% CI 66, 86%** | **Sp = 83% 95% CI 78, 87%** |  |  |

PPV = Positive Predictive Value; NPV = Negative Predictive Value; CI = Confidence Interval; Sn = sensitivity; Sp = specificity

* the components of the effectiveness algorithm are shown in Table 1

*** defined as (DAS28 <= 3.2) and high adherence (e.g. >= 80%) to the biologic started on the index date. Change in DAS28 could not be assessed since not all patients had a baseline VARA visit.

**Appendix Table B: Sensitivity Analysis Comparing the Effectiveness Algorithm* to a Different Definition of the Effectiveness Gold Standard** for Biologic and non-biologic DMARD* Users**

|  | **Met Effectiveness Gold Standard*** (DAS28 <= 3.2)** | | | |  |
| --- | --- | --- | --- | --- | --- |
| **Met Effectiveness Rule** |  | **Yes** | **No** | **Total** |  |
|  | **Yes** | 91 | 96 | 187 (27%) | **PPV = 49%,  95% CI 45, 53%** |
|  | **No** | 40 | 472 | 512(71%) | **NPV = 92%,**  **95% CI 91, 93%** |
|  | **Total** | 131(19%) | 568 (81%) | 699(100%) |  |
|  |  | **Sn = 69% 95% CI 65, 73%** | **Sp = 83% 95% CI 81, 85%** |  |  |

PPV = Positive Predictive Value; NPV = Negative Predictive Value; CI = Confidence Interval; Sn = sensitivity; Sp = specificity

* the components of the effectiveness algorithm are shown in Table 1

** includes hydroxychloroquine, leflunomide, and sulfasalazine

*** defined as (DAS28 <= 3.2) and high adherence (e.g. >= 80%) to the biologic/DMARD started on the index date. Change in DAS28 could not be assessed since not all patients had a baseline VARA visit.
